# Clinical evaluation of the aperture shape controller in volumetric modulated arc therapy: Effects on MLC and jaw motion complexity, plan quality, and deliverability

**DOI:** 10.1002/acm2.70215

**Published:** 2025-08-24

**Authors:** Tatsuya Kamima, Yosuke Sato, Yuki Murakami, Taro Takahashi, Masahiro Kaneko, Shiori Watanabe, Hikaru Miyauchi, Natsumi Abo, Yasushi Ito, Takashi Toshiyasu, Senzo Taguchi, Arisa Harada, Yasuo Yoshioka

**Affiliations:** ^1^ Radiation Oncology Department Cancer Institute Hospital Japanese Foundation for Cancer Research Koto‐ku Tokyo Japan

**Keywords:** Aperture shape controller, Jaw tracking, JTCS, MCSv, VMAT

## Abstract

**Background:**

This study comprehensively evaluated the impact of aperture shape controller (ASC) on volumetric modulated arc therapy (VMAT) clinical treatment planning.

**Methods:**

A total of 248 VMAT plans for head and neck, prostate, post mastectomy breast, gastric MALT lymphoma, rectum, and Lung SBRT for treatments performed between 2018 and 2023 were retrospectively enrolled. These treatment plans were divided into two groups: before (ASC‐OFF group) and after (ASC‐ON group) the clinical implementation of ASC. The performance of ASC in terms of plan complexity was evaluated by between group comparisons of the multi‐leaf collimator (MLC) modulation complexity score (MCS_v_) and jaw tracking complexity score (JTCS), plan quality using dosimetric parameters, and plan deliverability.

**Results:**

The overall MCS_v_ and JTCS of the ASC‐OFF and ASC‐ON groups were 0.318 and 0.369 (*p *< 0.001) and 2.85 cm and 2.98 cm (*p* = 0.10), respectively. In the ASC‐ON group, the complexity of MLC motions was significantly lower and the complexity of jaw motions higher. In addition, most dosimetric parameters were comparable between groups or better in the ASC‐ON group. There was no significant difference in the gamma passing rate of 3%/2 mm between the two groups, although at tighter criteria, the gamma passing rate was higher in the ASC‐ON group.

**Conclusions:**

The use of ASC reduced the complexity of MLC motions without compromising the plan quality. Furthermore, this study found that ASC allows jaw tracking to be more effective.

## 1 INTRODUCTION

Volumetric modulated arc therapy (VMAT) provides a highly conformal dose distribution with reduced unnecessary dose to the surrounding normal tissue by using simultaneous modulation of the multileaf collimator (MLC) aperture. While high intensity modulation, including a complex MLC aperture shape, can obtain an ideal dose distribution, it leads to increased monitor unit (MU).^1^, ^2^ Studies have also reported that with the frequent use of small or irregular fields with relatively low dose‐calculation accuracy, there might be a clinically significant discrepancy between the dose calculated by the radiotherapy treatment planning system (TPS) and its actual delivery in highly modulated VMAT plans.^1^, ^3^, ^4^, ^5^, ^6^

The aperture shape controller (ASC) is an optimization tool that controls the MLC aperture size and shape in the Photon Optimizer algorithm of the Eclipse TPS (Varian Medical Systems, Palo Alto CA, USA). The ASC penalizes disconnected apertures created by adjacent leaf pairs. There are six settings: None (no constraints), Very low, Low, Moderate, High, and Very high (maximum constraints). The higher the ASC setting, the more strongly the optimization algorithm pushes to join adjacent apertures, reducing the complexity of the treatment plan.^7^ Several previous reports showed that the ASC reduced the complexity of the MLC aperture shape without compromising the plan quality.^7^, ^8^, ^9^, ^10^ Furthermore, Scaggion et al. showed that use of the ASC increases plan deliverability: compared with treatment planning without the ASC setting, gamma passing rates in dosimetric measurements were significantly increased in prostate and oropharyngeal cancers.^11^

Conversely, the reports on ASC are also subject to some limitations in their considerations. In most of the reports evaluating ASCs, the original plans were copied and applied by changing only the ASC settings and reoptimizing with all other objectives and settings held constant, and no user interaction.^7^, ^8^, ^9^, ^10^, ^11^ However, in practice, the plan optimization process is performed by determining the objective function and priorities using trial and error iteration. Studies have reported that these manual interventions affect the quality of treatment planning, and that the complexity of MLC motions may change significantly.^12^, ^13^, ^14^, ^15^ Therefore, the effectiveness of ASC in terms of clinical treatment planning is still unclear. To clarify this, it is necessary to compare treatment plans with or without ASCs used in clinical practice.

In recent years, the linear accelerator of a TrueBeam system (Varian Medical Systems, Palo Alto CA, USA) with a jaw tracking (JT) technique has been widely used in VMAT, and with this, both the MLC and the jaw motion are complicated.^16^, ^17^ The JT technique can minimize the leakage and transmission through the MLC by making the jaws follow the MLC apertures. The impact of ASC on the complexity of jaw motion has also not yet been investigated.

This study compared treatment planning used in clinical practice before and after the implementation of ASC. The purpose of this study was to determine the effectiveness of ASC in clinical treatment planning in terms of (1) the complexity of the MLC and jaw motions, (2) plan quality, and (3) plan deliverability.

## 2 METHODS

### 2.1 Patient selection

In this study, a total of 248 clinical treatment plans were retrospectively selected, including patients treated between January 2018 and January 2023 for head and neck (H&N) cancer (oropharyngeal, hypopharyngeal, or gingival), localized prostate cancer, left‐sided postmastectomy breast cancer, gastric mucosa‐associated lymphoid tissue (gMALT) lymphoma, rectal cancer, or peripheral lung cancer treated with stereotactic body radiotherapy (SBRT). These anatomical regions were selected to account for variations in target shape, volume, and anatomical complexity. Patients were selected based on consistent criteria, including the number of arcs, gantry angle range, prescription dose, margin size, and dose constraints, considering their influence on treatment planning complexity. Table 1 shows the details of the cases and VMAT planning parameters at each anatomical site. In February 2020, the ASC default setting in the TPS was changed to Moderate, and ASC was implemented for clinical use in our institution. Therefore, cases treated between January 2018 and January 2020, the period before the implementation of ASC, were defined as the ASC‐OFF group, and cases treated between February 2020 and January 2023, the period with implementation of ASC, were defined as the ASC‐ON group. Written informed consent was obtained from all patients, and the institutional ethics committee approved this study (review board number: 2021‐GA‐1038).

**TABLE 1:** Case details and planning parameters.

	H&N	Prostate	PM breast	gMALT	Rectum	Lung SBRT
Cases (ASC‐OFF/ON)						
Number	25/26	21/29	22/21	21/21	11/30	7/14
Average PTV volume (cm^3^)	684.6/780.5	76.9/82.2	1026.5/1040.4	1331.2/995.5	791.0/872/0	22.0/15.5
Treatment planners						
Number	2	2	4	2	3	2
Planning parameters						
Prescription						
Total dose (Gy)	66	70	50	30.6	50	55
Fraction dose (Gy)	2	2.5	2	1.8	2	4
Beam parameter						
Beam energy (MV)	6‐FF	10‐FF	10‐FF	10‐FF	10‐FF	6‐FFF
Number of arcs	2	1	2	2	2	2
Gantry rotation	181°–179° 179°–181°	181°–179°	300°–179° 179°–300°	230°–179° 179°–230°	240°–120° 120°–240°	330° (±20°)‐179° 179°‐330°(± 20°) 30° (±20°)‐181° 181°–30°(± 20°)
Collimator rotation	20° (±10°) 340° (±10°)	30°	15° 345°	30° 330°	15° 345°	20° 340°

Abbreviations: FFF, flattening filter‐free; gMALT, gastric mucosa‐associated lymphoid tissue lymphoma; H&N, head and neck; PM breast, postmastectomy breast; PTV, planning target volume; SBRT, stereotactic body radiotherapy.

### 2.2 Treatment planning

All VMAT plans were created using an Eclipse TPS Ver.15.6 and were delivered using a TrueBeam linear accelerator with a Millennium 120 Leaf MLC. For each case, a CT‐scan was acquired with 2.0‐mm slice thickness and 50‐cm field of view. These plans used a maximum dose rate of 600 MU/min, and the JT technique was enabled.

In optimization process, photon optimizer Ver.15.6 was used for this study. The plans were created by expert planners per anatomical regions, through trial‐and‐error iterations to achieve the dose constraints shown in Table S1. There were no major changes in dose constraints at any anatomical sites. In the ASC‐ON group, if the clinically acceptable plan could not be achieved by adjusting the optimization parameters, the ASC setting was basically changed from Moderate to a weaker constraint level. Additionally, the maximum MU setting was not applied during the treatment planning process in this study. For dose calculation, the anisotropic analytic algorithm was used with a dose calculation grid size of 2.5 mm. All clinical treatment plans underwent a two‐step review process: initial verification by a specific medical physicist, followed by final approval by a specific radiation oncologist assigned to each anatomical site.

### 2.3 Multileaf collimator and jaw motion complexity

The complexity of the MLC aperture shape for ASC‐OFF and ASC‐ON groups was quantified using the modulation complexity score for VMAT (MCS_V_) proposed by Masi et al.^4^ Although there are many indices of plan complexity,^18^ MCS_V_ was adopted in this study because previous reports have been able to detect a reduction in complexity of the MLC aperture shape according to the ASC level.^7^, ^10^, ^19^ The MCS_V_ has values in the range from 0 to 1. When the modulation complexity of a VMAT plan MLC motion increases, the value of MCS_V_ decreases.

The complexity of jaw motions for ASC‐OFF and ASC‐ON groups was also quantified using the JT complexity score (JTCS) [cm] proposed by Murakami et al.^17^ A larger JTCS value indicates more complex jaw motions during JT. All plans were exported as DICOM‐RT plan files. An in‐house application developed in Python (Ver. 3.6.4) was then applied to these DICOM‐RT plan files to calculate the MCS_V_, JTCS, and MU/cGy.

### 2.4 Evaluation of plan quality

The following indices were used to compare the dosimetric parameters of the target and OARs between the ASC‐OFF and ASC‐ON groups. For planning target volumes (PTV) or clinical target volumes, the maximum dose (dose received by 2% of the target volume, D_2%_), minimum dose (dose received by 98% of the target volume, D_98%_), and dose received by 50% of the target volume (D_50%_) were analyzed. The main OARs at each anatomical site were evaluated using the dosimetric parameters shown in Table 2. *V_x_* represents the volume receiving no less than *x*% of the prescription dose. *D_x_* represents the minimum dose delivered to *x*% of the structure volume.

**TABLE 2:** Dosimetric parameters of the OARs at each anatomical site.

Anatomical site	Objects	Dosimetric parameter
H&N	Brain stem	D_max_ (Gy)
	Spinal cord	D_max_ (Gy)
	Parotid gland right	D_mean_ (Gy)
	Parotid gland left	D_mean_ (Gy)
Prostate	Rectum wall	D_2%_ (%), D_20%_ (%), D_40%_ (%), D_60%_ (%)
	Bladder wall	D_2%_ (%), D_20%_ (%), D_40%_ (%), D_60%_ (%)
PM breast	Lung left	V_20_ (%), V_5_ (%)
	Heart	D_mean_ (Gy)
	Spinal cord	D_max_ (Gy)
gMALT	Heart	D_mean_ (Gy)
	Kidney right	V_10_ (%)
	Kidney left	V_10_ (%)
	Liver	V_20_ (%)
	Spinal cord	D_max_ (Gy)
Rectum	Large bowel	V_30_ (%), V_10_ (%)
	Small bowel	V_30_ (%), V_10_ (%)
	Bladder	V_40_ (%)
	Bone marrow	V_40_ (%), V_20_ (%)
	Femoral head right	V_30_ (%)
	Femoral head left	V_30_ (%)
Lung SBRT	Lungs‐GTV	D_mean_ (Gy), V_20_ (%), V_15_ (%)

Abbreviations: Dx, minimum dose delivered to x% of the structure volume; gMALT, gastric mucosa‐associated lymphoid tissue lymphoma; H&N, head and neck; OAR, organs at risk; PM breast, postmastectomy breast; SBRT, stereotactic body radiotherapy; Vx, volume receiving no less than x% of the prescription dose.

In postmastectomy breast, a virtual CT dataset (CT_virtual) based on the skin flash methods was created by adding a virtual bolus to the skin surface in the region where the PTV was contoured, to compensate for the dose to the skin surface and low positional uncertainty.^20^, ^21^ Thereafter, zero Hounsfield units were assigned to the expanded region. CT_virtual was used only for the optimization processes, and after the optimization, the VMAT plans using CT_virtual were copied to the original CT datasets (CT_original) and recalculated by fixing the MU. For the dosimetric evaluation, a plan with dose calculations performed for CT_original was used.

### 2.5 Evaluation of plan deliverability

Patient‐specific quality assurance (PSQA) was performed for all clinical treatment planning using the Delta4 device (ScandiDos, Uppsala, Sweden). For the calculation of reference dose distributions, a 2.5 mm calculation grid size was used. Before the clinical plan measurements, box‐shaped dose distributions were measured from four 10 × 10‐cm fields (45°, 135°, 225°, 315°). The four‐field box distribution was chosen to reduce uncertainty introduced in the measurement by set‐up errors. The position of the Delta4 was adjusted in the application so that the measured dose distribution matched the four‐field box distribution calculated for the Delta4 in the TPS. The measured dose at the central detector was compared with the calculated dose to obtain the daily output correction factor, which was then applied to the clinical plan measurements. These correction factors were used to account for variations in the output of the radiotherapy machine. The measurements by the true composite method were compared with the predicted values of the TPS using the global gamma passing rate following evaluation criteria: 3% dose difference and 2 mm distance to agreement (3%/2 mm), 2%/2, 2%/1, and 1%/1 mm. The low dose exclusion threshold was 10% relative to the maximum dose of the Delta4 phantom plan.

### 2.6 Statistical analysis

The differences in six anatomical site plans between the ASC‐OFF and ASC‐ON groups were evaluated using the Mann‐Whitney U‐test for the main evaluation metrics, MCS_v_ and JTCS, in this study. Concerning multiple testing of ASC‐OFF and ASC‐ON groups, we conservatively adjusted the p‐values after the Bonferroni correction (*p* = 0.05/10 = 0.005). All statistical analyses were conducted with JMP 15.1.0 (SAS Institute, Cary, NC, USA).

## 3 RESULTS

Table 3 shows the ASC settings selected in the ASC‐ON group. The most‐selected ASC setting in all anatomical sites was the default setting of Moderate.

**TABLE 3:** Proportions of ASC settings selected in the ASC‐ON group.

Anatomical site	H&N	Prostate	PM breast	gMALT	Rectum	Lung SBRT
ASC level						
Very high	0%	0%	0%	0%	0%	0%
High	0%	0%	10%	0%	0%	0%
Moderate	81%	100%	86%	86%	87%	100%
Low	4%	0%	4%	4%	0%	0%
Very low	15%	0%	0%	10%	13%	0%

Abbreviations: ASC, aperture shape controller; gMALT, gastric mucosa‐associated lymphoid tissue lymphoma; H&N, head and neck; PM breast, postmastectomy breast; SBRT, stereotactic body radiotherapy.

**FIGURE 1:**
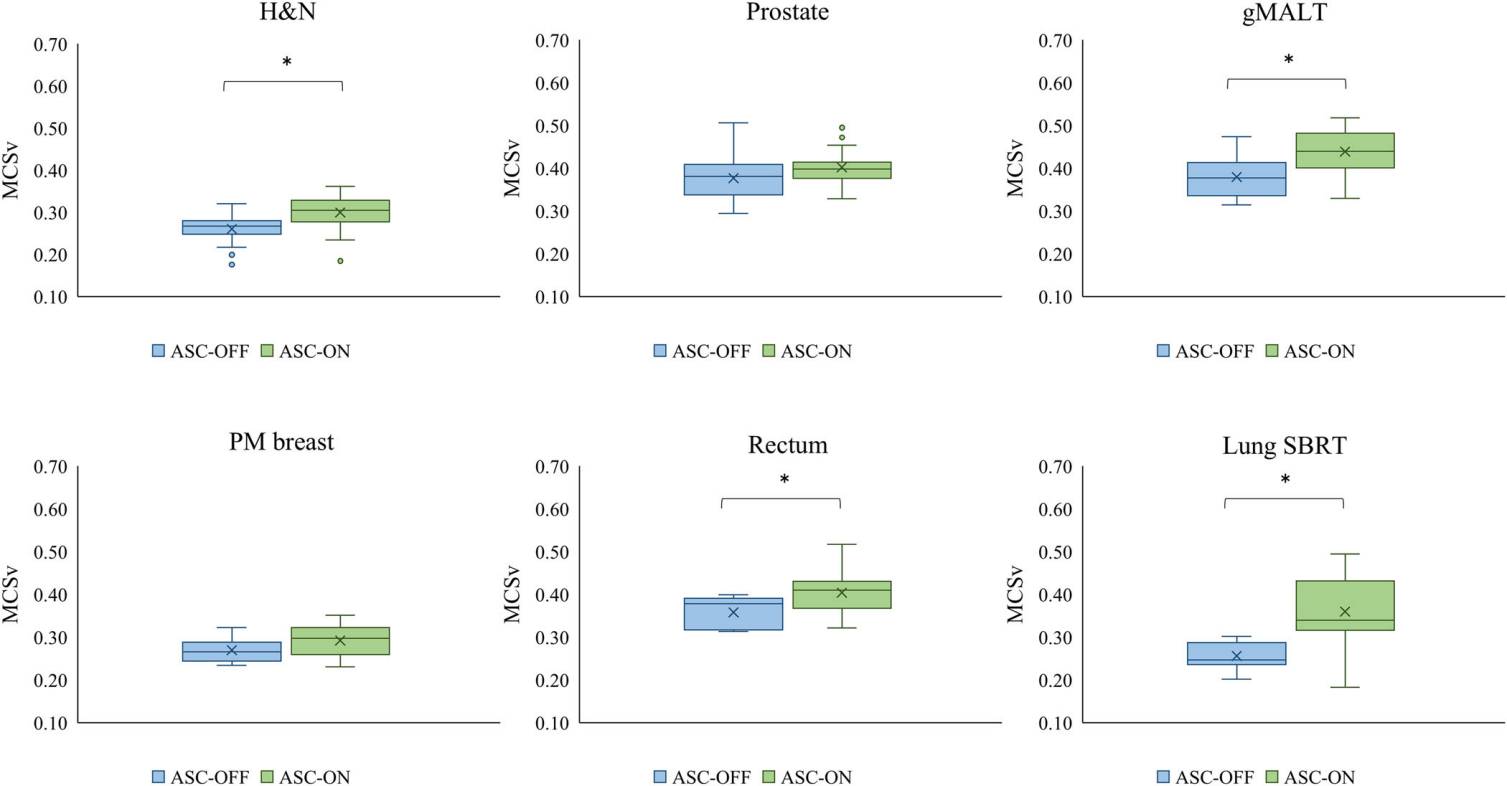
Box‐and‐whisker plots showing the MCS_v_ of ASC‐OFF and ASC‐ON groups at each anatomical site. The upper and lower edges represent the 25th and 75th percentiles, respectively. Whiskers represent the standard deviation. Outliers are marked with circles and were defined according to 1.5× the interquartile range. *p < 0.005.

The overall MCS_v_ values of the ASC‐OFF and ASC‐ON groups were 0.318 and 0.369 (*p *< 0.001), respectively. Figure 1 shows box‐and‐whisker plots of the MCS_v_ of ASC‐OFF and ASC‐ON groups at each anatomical site. The MCS_v_ was higher in the ASC‐ON group than in the ASC‐OFF group, except for treatment to the prostate. The use of ASC decreased the complexity of MLC motions.

**FIGURE 2:**
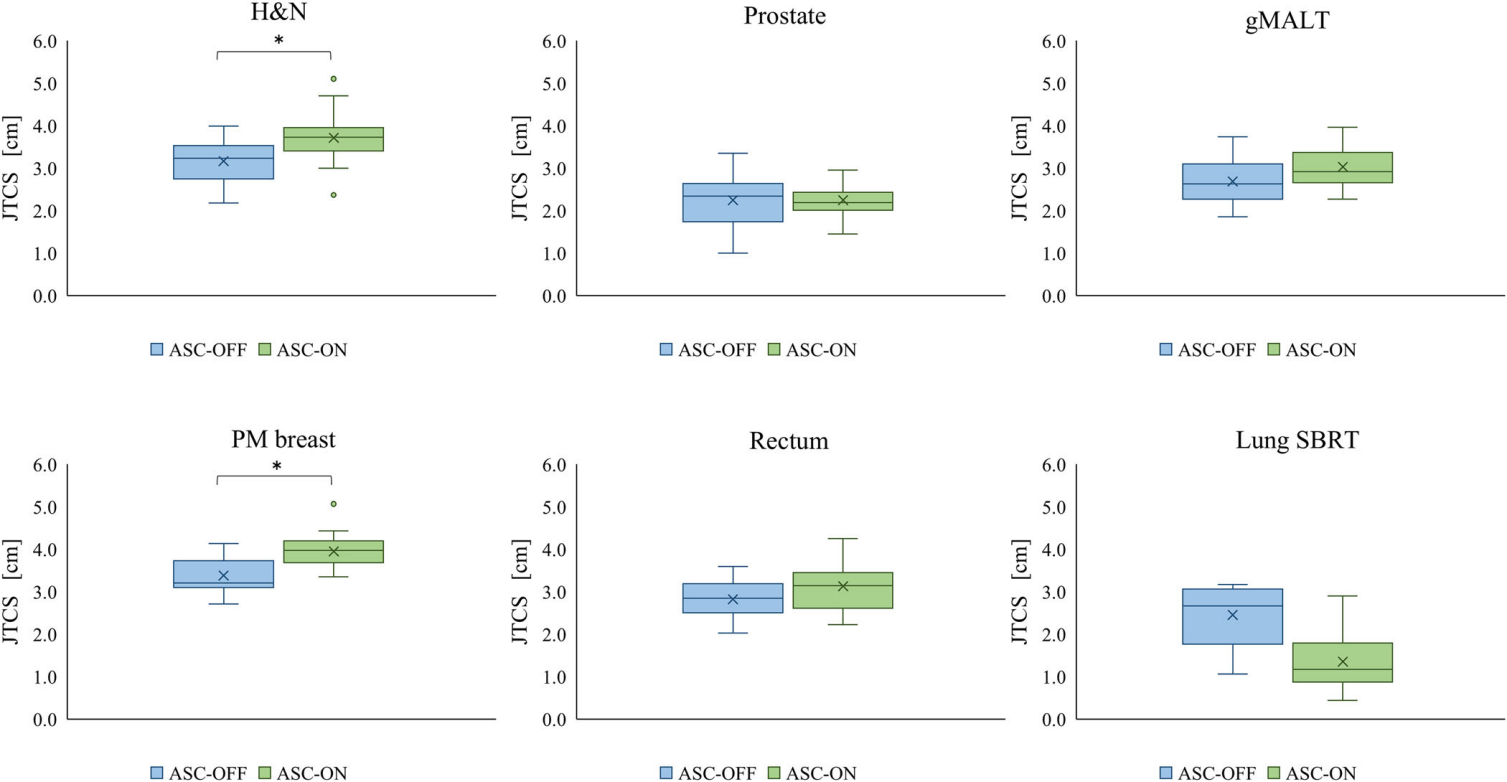
Box‐and‐whisker plots showing the JTCS of ASC‐OFF and ASC‐ON groups at each anatomical site. The upper and lower edges represent the 25th and 75th percentiles, respectively. Whiskers represent the standard deviation. Outliers are marked with circles and were defined according to 1.5× the interquartile range. *p < 0.005.

The overall JTCS values of the ASC‐OFF and ASC‐ON groups were 2.85 cm and 2.98 cm (*p* = 0.10), respectively. Figure 2 shows box‐and‐whisker plots of the JTCS of the ASC‐OFF and ASC‐ON groups for each anatomical site. JTCS was higher in the ASC‐ON group than in the ASC‐OFF group, except for the prostate and lung SBRT. The use of ASC increased the complexity of jaw motions during JT.

Table 4 summarizes the average MU/cGy in the ASC‐OFF and ASC‐ON groups. The MU/cGy were higher in the ASC‐OFF than ASC‐ON condition in the gMALT and rectum, and lung SBRT, but there were no differences in other sites.

**TABLE 4:** Summary of the average MU/cGy in the ASC‐OFF and ASC‐ON groups.

	Average MU/cGy (95% CI)	
Anatomical site	ASC‐OFF	ASC‐ON
H&N	2.79 (2.67–2.90)	2.77 (2.66–2.87)
Prostate	2.68 (2.60–2.76)	2.70 (2.64–2.77)
PM breast	2.58 (2.49–2.68)	2.54 (2.41–2.66)
gMALT	2.66 (2.57–2.76)	2.19 (2.10–2.28)
Rectum	2.31 (2.20–2.41)	2.15 (2.08–2.22)
Lung SBRT	3.54 (3.16–3.92)	2.87 (2.73–3.02)

Abbreviations: ASC, aperture shape controller; CI, confidence interval; gMALT, gastric mucosa‐associated lymphoid tissue lymphoma; H&N, head and neck; MU, monitor unit; PM breast, postmastectomy breast; SBRT, stereotactic body radiotherapy.

A summary of the dosimetric parameters is listed in Table 5. Compared with the ASC‐OFF group, the dosimetric parameters in the ASC‐ON group were similar or improved.

**TABLE 5:** Summary of the dosimetric parameters in the ASC‐OFF and ASC‐ON groups.

			ASC‐OFF	ASC‐ON
Anatomical site	Objects	Criteria	Average (95% CI)	
H&N	PTV_66	D_2%_ (%)	106.3 (106.7–107.8)	105.3(105.8–106.5)
		D_50%_ (%)	104.5 (104.1–104.9)	103.8 (103.5–104.1)
		D_98%_ (%)	97.8 (97.3–98.2)	97.2 (96.4–98.1)
	PTV_54	D_2%_ (%)	95.4 (94.1–96.6)	96.0 (95.1–96.9)
		D_50%_ (%)	85.5 (85.0–86.1)	85.6 (85.3‐85.9)
		D_98%_ (%)	73.9 (73.0–74.9)	75.2 (74.5–76.0)
	Brain stem	D_max_ (Gy)	18.8 (16.8–20.9)	20.5 (17.7–23.3)
	Spinal cord	D_max_ (Gy)	41.8 (40.6–42.9)	39.4 (37.9‐40.8)
	Parotid gland right	D_mean_ (Gy)	25.8 (22.2–29.3)	25.2 (22.1–28.3)
	Parotid gland left	D_mean_ (Gy)	26.5 (24.1–28.8)	25.5 (21.5–29.5)
Prostate	PTV	D_2%_ (%)	104.1 (103.8–104.3)	103.8 (103.6–104.0)
		D_50%_ (%)	100.8 (100.6–101.1)	100.9 (100.8–101.1)
		D_98%_ (%)	88.6 (87.9–89.3)	89.0 (88.7–89.3)
	Rectum wall	D_2%_ (%)	95.4 (92.9–97.8)	96.3 (93.9–98.7)
		D_20%_ (%)	67.1 (62.3–72.0)	71.0 (66.6–75.4)
		D_40%_ (%)	37.7 (34.5–41.0)	38.1 (36.2–40.1)
		D_60%_ (%)	22.4 (20.0–24.7)	22.2 (21.3–23.2)
	Bladder wall	D_2%_ (%)	98.9 (98.5–99.3)	99.4 (99.1–99.7)
		D_20%_ (%)	67.3 (60.4–74.2)	62.7 (56.9–68.5)
		D_40%_ (%)	34.2 (27.7–40.7)	24.8 (19.4–30.2)
		D_60%_ (%)	16.8 (11.7–21.9)	10.2 (7.2–13.2)
PM breast	CTV	D_2%_ (%)	108.2 (107.7–108.7)	106.7 (106.2–107.3)
		D_50%_ (%)	104.3 (103.9–104.8)	102.9 (102.4–103.4)
		D_98%_ (%)	99.0 (98.4–99.7)	97.5 (96.7–98.3)
	Lung left	V_5_ (%)	74.4 (72.4–76.5)	65.2 (61.3–69.2)
		V_20_ (%)	31.7 (30.1–33.4)	26.7 (23.6–29.8)
	Heart	D_mean_ (Gy)	8.2 (7.9–8.5)	7.6 (7.1–8.1)
	Spinal cord	D_max_ (Gy)	19.4 (18.4–20.4)	12.7 (12.1–13.3)
gMALT	PTV	D_2%_ (%)	105.2 (104.9–105.5)	104.2 (103.9–104.5)
		D_50%_ (%)	101.7 (101.5–101.8)	102.0 (101.7–102.2)
		D_98%_ (%)	91.1 (89.7–92.6)	93.1 (92.4–93.7)
	Heart	D_mean_ (Gy)	17.0 (12.8–21.3)	8.3 (6.6–10.0)
	Spinal cord	D_max_ (Gy)	19.3 (18.3–20.4)	15.6 (15.0–16.1)
	Kidney right	V_10_ (%)	15.3 (8.4–22.2)	3.8 (1.4–6.1)
	Kidney left	V_10_ (%)	24.6 (16.7–32.6)	16.5 (8.3–24.6)
	Liver	V_20_ (%)	24.6 (21.0–28.3)	16.8 (14.7–18.9)
Rectum	PTV	D_2%_ (%)	104.3 (104.1–104.5)	104.1 (103.9–104.3)
		D_50%_ (%)	101.9 (101.7–102.0)	102.0 (101.8–102.1)
		D_98%_ (%)	95.6 (95.2–95.9)	95.8 (95.5–96.2)
	Small bowel	V_10_ (%)	48.4 (35.3–61.4)	44.3 (36.5–52.1)
		V_30_ (%)	11.7 (5.7–17.7)	5.5 (3.3–7.6)
	Large bowel	V_10_ (%)	50.6 (40.9–60.3)	58.4 (51.6–65.3)
		V_30_ (%)	19.1 (11.7–26.6)	21.8 (17.5–26.0)
	Bladder	V_40_ (%)	18.3 (11.2–25.4)	17.7 (11.9–23.5)
	Bone Marrow	V_20_ (%)	51.6 (47.7–55.6)	52.3 (50.0–54.6)
		V_40_ (%)	17.7 (15.9–19.4)	15.2 (14.0–16.3)
	Femoral head right	V_30_ (%)	3.2 (1.9–4.4)	1.9 (1.2–2.6)
	Femoral head left	V_30_ (%)	4.2 (2.5–5.9)	2.9 (2.1–3.7)
Lung SBRT	PTV	D_2%_ (%)	127.7 (104.9–105.5)	127.0 (103.9–104.5)
		D_50%_ (%)	115.2 (112.6–117.8)	114.8 (113.7–115.9)
		D_98%_ (%)	97.6 (97.1–98.1)	96.7 (95.0–98.4)
	Lungs‐GTV	D_mean_ (Gy)	2.2 (1.3–3.2)	2.4 (2.0–2.8)
		V_15_ (%)	3.6 (1.9–5.3)	4.2 (3.2–5.3)
		V_20_ (%)	2.4 (1.3–3.4)	2.8 (2.0–3.5)

Abbreviations: CI, confidence interval; CTV, clinical target volume; Dx, the minimum dose delivered to x% of the structure volume; gMALT, gastric mucosa‐associated lymphoid tissue lymphoma; H&N, head and neck; PM breast, postmastectomy breast; PTV, planning target volume; SBRT, stereotactic body radiotherapy; Vx, the volume receiving no less than x% of the prescription dose.

**FIGURE 3:**
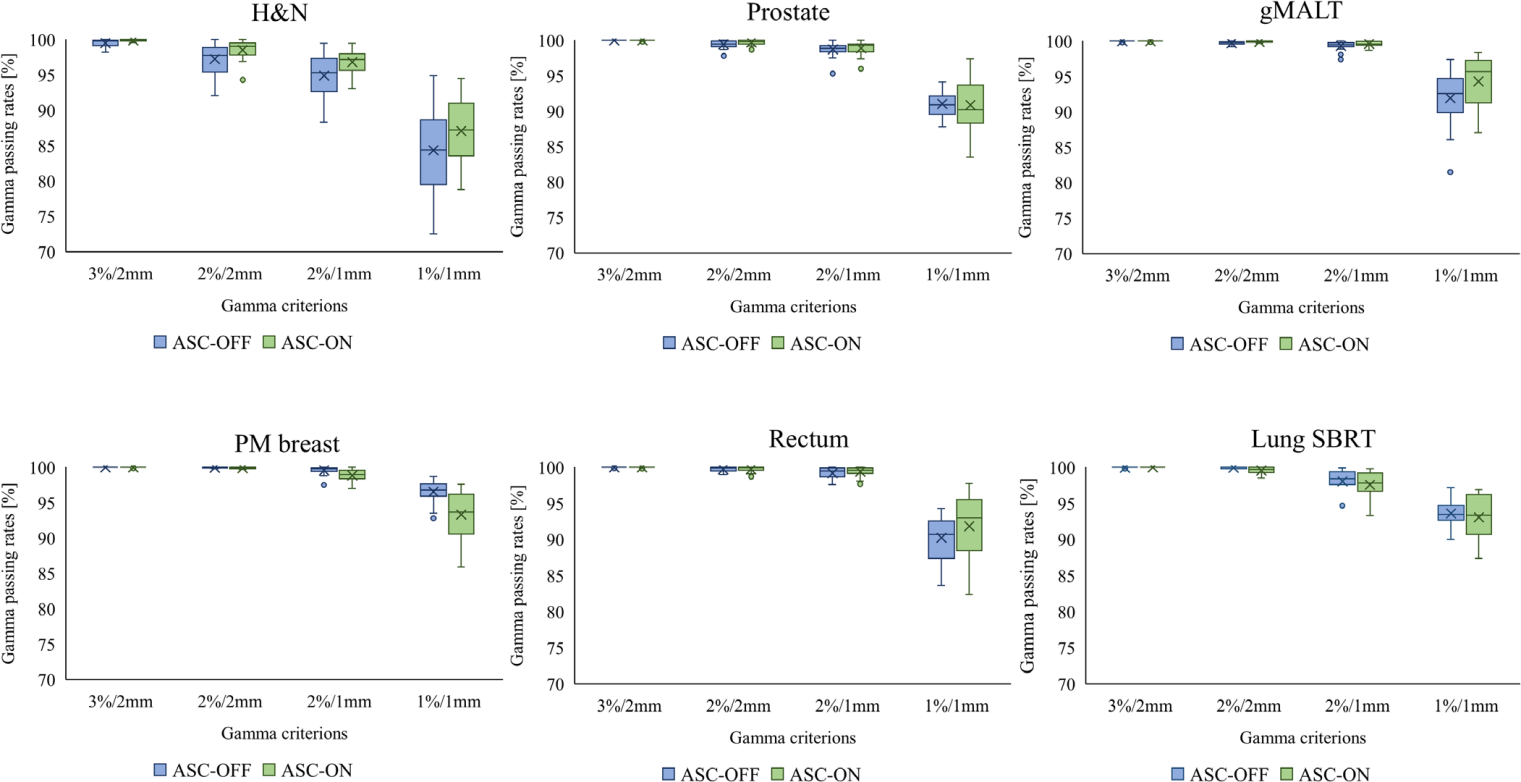
Box and whisker plots showing the gamma passing rates of ASC‐OFF and ASC‐ON groups at each anatomical site. The upper and lower edges represent the 25th and 75th percentiles, respectively. Whiskers represent the standard deviation. Outliers are marked with circles and were defined according to 1.5× the interquartile range.

Box‐and‐whisker plots of the Delta4‐measured gamma passing rates for the VMAT plans for each anatomical site are shown in Figure 3. There was no difference in the gamma passing rate with 3%/2 mm between the ASC‐OFF and ASC‐ON groups for most anatomical sites, except for the H&N. In the H&N, the gamma passing rate of the ASC‐ON group was better than that of the ASC‐OFF group. Furthermore, in most other sites, the gamma passing rate at tighter criteria was higher in the ASC‐ON group than in the ASC‐OFF group.

## 4 DISCUSSION

This study investigated the impact of ASC on treatment planning using 248 clinical plans for six anatomical sites. Although there have been several reports on the usefulness of ASC in each anatomical site, no evaluation of ASC has included trial‐and‐error iteration in the optimization process and included such a large number of cases. This parameter tuning iteration is an essential part of the process for creating the patient's clinical treatment planning. It is also widely recognized as having a significant impact on the quality of the treatment planning.^12^, ^13^, ^14^, ^15^ In general, the creation of steeper dose distributions through iterative parameter tuning increases the complexity of the MLC motion and the MU. In this study, despite the inclusion of such clinical treatment planning, the ASC decreased the complexity of the MLC motion at most anatomical sites, as shown in Figure 1. In addition, as shown in Table 5, ASC did not worsen the plan quality. Thus, the results of this study indicate the usefulness of ASC in clinical treatment planning.

The JT continuously adjusts the main jaw to tangentially enclose the distal apertures shaped by the MLC.^22^ As shown in Figure 2, ASC increased the complexity of jaw motion, with the JTCS values of the ASC‐ON group being significantly higher than those of the ASC‐OFF group, except for the lung SBRT. The ASC acts through the penalty term of the total cost function and attempts to join the disconnected apertures defined by pairs of adjacent leaves.^11^ Therefore, in the ASC‐ON group, the jaws effectively tracked the MLC, because the MLC tip positions were not separated and adjacent leaf pairs moved less independently than ASC‐OFF group. One concern is the impact of the complexity of the jaw motion on jaw position accuracy and dose variation. Matsubayashi et al. investigated the influence of jaw position accuracy on dose variation in VMAT with the JT technique, and reported that the maximum dose variations were 0.179% and 1.23% when the errors were 1 and 10 mm, respectively.^23^ Therefore, the dose variation due to jaw position error caused by complex motions is smaller than that due to MLC position error and is clinically negligible. As shown in Table 1, the PTV was substantially smaller in lung SBRT compared to other anatomical sites. For such small targets, the ASC reduces the complexity of MLC motion, allowing the MLC to remain open to the PTV for most segments. Therefore, we consider that the ASC decreased the complexity of jaw motion in lung SBRT.

As Figure 1 shows, ASC appeared to have less effect on the MLC motions in the prostate. Scaggion et al. similarly reported that ASC was less effective at reducing the complexity of MLC motions in prostate compared with the H&N region.^11^ In their report, they explained that this was due to differences in anatomical complexity, and that the impact of ASC may be limited in the prostate because MLC shaping is inherently simpler. In this study, the ASC setting was not changed to a stronger ASC constraint in the prostate, as achieving dose constraint was the top priority. Ito et al. reported that for prostate plans, the complexity of MLC motion can be reduced by using the Very high setting without causing deterioration in plan quality.^19^ Therefore, for relatively simple MLC motion plans, such as those for prostate, a stronger ASC constraint setting may be more appropriate than the Moderate setting.

The Task Group 218 (TG‐218) report published by the American Association of Physicists in Medicine recommended the use of a 3%/2 mm criterion for gamma index analysis to evaluate PSQA.^24^ As Figure 3 shows, the effects on plan deliverability of the use and non‐use of ASC may not be detectable in PSQA. However, several studies reported that minor MLC positioning errors can be detected with high sensitivity when tighter gamma criterions are used.^25^, ^26^, ^27^, ^28^ When the criterion was tightened, as shown in Figure 3, the ASC‐ON group showed an improvement in gamma passing rate in many anatomic regions, which may have improved the accuracy of MLC motion. Such an accurate agreement between the calculated dose and the actual delivery dose with improved MLC motion is very important in safe delivery of irradiation. Therefore, the improved plan deliverability obtained with the use of ASC is a clinical advantage.

Our study had several limitations. Our objective was to evaluate the impact of ASC in a more clinically realistic setting—namely, by determining the objective function and priorities through an iterative trial‐and‐error planning process. Accordingly, it was necessary to use different patient cohorts in this study. Therefore, individual anatomical differences between patients and other confounding factors could not be completely eliminated, and the results may have been influenced by these factors. Furthermore, as this study was conducted over a 5‐year period, multiple expert planners were involved in the creation of the treatment plans. Studies have reported that the plan quality of VMAT plans, which are created by inverse planning, depends on the planner's experience and skills.^13^, ^29^, ^30^ However, the final approval of the treatment planning during this study was performed only by specific radiation oncologists and medical physicist with specialism in each anatomical site, and consistency was therefore maintained in dose distribution and adherence to clinical dose constraints.

## 5 CONCLUSION

In the present study, we comprehensively evaluated the impact of ASC on treatment planning using a clinical treatment plan that included trial and error iteration in the optimization process. In the ASC‐ON group, the complexity of the MLC motions was lower and the jaw motions were more effective than in the ASC‐OFF group. Moreover, the ASC‐ON group had a higher gamma passing rate in the tighter criteria, which improved plan deliverability. These findings indicate the usefulness of ASC for clinical treatment planning.

## AUTHOR CONTRIBUTIONS

**Tatsuya Kamima**: Conceptualization; methodology; writing—original draft preparation. **Yosuke Sato**: Software; resources. **Yuki Murakami**: Formal analysis. **Taro Takahashi**: Resources. **Masahiro Kaneko**: Resources. **Shiori Watanabe**: Resources. **Hikaru Miyauchi**: Resources. **Natsumi Abo**: Resources. **Yasushi Ito**: Resources; project administration. **Takashi Toshiyasu**: Resources. **Senzo Taguchi**: Resources. **Arisa Harada**: Resources. **Yasuo Yoshioka**: Project administration; writing—review & editing; funding acquisition.

## CONFLICT OF INTEREST STATEMENT

The authors declare no conflicts of interest.

## ETHICAL STATEMENT

2021‐GA‐1038.

## Supporting information



Supporting Information
