# Supplementary material for: Clinical evaluation of the aperture shape controller in volumetric modulated arc therapy: Effects on MLC and jaw motion complexity, plan quality, and deliverability
Source: J Appl Clin Med Phys. 2025 Aug 24;26(9):e70215. doi: 10.1002/acm2.70215 (PMC12375282; doi:10.1002/acm2.70215)
Supplement: Supplementary file 1 — Supporting Information [file ACM2-26-e70215-s001.docx]

**Supplementary Material**

**Clinical Evaluation of the Aperture Shape Controller in Volumetric Modulated Arc Therapy: Effects on MLC and Jaw Motion Complexity, Plan Quality, and Deliverability**

**Supplementary Table 1.** Dose constraints for treatment planning at each anatomical site

| Anatomical site | Organ |  |  | Target |  |
| --- | --- | --- | --- | --- | --- |
| H&N | Brain stem | D_max_ < 54.0 Gy |  | PTV_66 | D_2%_ < 120.0% |
|  |  | D_1cm³_ < 60.0 Gy |  |  | D_10%_ < 110.0% |
|  | Spinal cord | D_max_ < 45.0 Gy |  |  | D_50%_ < 105.0% |
|  |  | D_1cm³_ < 50.0 Gy |  |  | D_95%_ ≥ 100.0% |
|  | Ear inner right | D_mean_ < 45.0 Gy |  |  | D_98%_ ≥ 93.0% |
|  | Ear inner left | D_mean_ < 45.0 Gy |  | PTV_54 | D_2%_ < 98.2% |
|  | Esophagus | D_mean_ < 30.0 Gy |  |  | D_10%_ < 90.0% |
|  | Parotid gland right | D_mean_ < 20.0 Gy |  |  | D_50%_ < 86.0% |
|  | Parotid gland left | D_mean_ < 20.0 Gy |  |  | D_95%_ ≥ 82.0% |
|  | Glnd thyroid | D_mean_ < 60.0 Gy |  |  | D_98%_ ≥ 76.1% |
|  | Larynx | D_max_ < 50.0 Gy |  |  |  |
|  |  | D_mean_ < 20.0 Gy |  |  |  |
|  | Lips | D_mean_ < 20.0 Gy |  |  |  |
|  | Mandible | D_max_ < 60.0 Gy |  |  |  |
|  |  | D_2cm³_ < 66.0 Gy |  |  |  |
|  | Oral cavity | D_1%_ < 110.0% |  |  |  |
|  | Skin | D_1%_ < 110.0% |  |  |  |
|  | Submandibular gland right | D_mean_ < 35.0 Gy |  |  |  |
|  | Submandibular gland left | D_mean_ < 35.0 Gy |  |  |  |
|  |  |  |  |  |  |
| Prostate | Rectum wall | D_2%_ < 99.4% |  | Prostate | D_99.9%_ ≥ 98.5% |
|  |  | D_20%_ < 88.2% |  |  | D_min_ ≥ 98.5% |
|  |  | D_40%_ < 52.9% |  | CTV | D_98%_ ≥ 99.9% |
|  |  | D_60%_ < 32.8% |  |  |  |
|  |  | D_80%_ < 21.7% |  |  |  |
|  | Bladder wall | D_2%_ < 101.4% |  |  |  |
|  |  | D_20%_ < 88.3% |  |  |  |
|  |  | D_40%_ < 53.2% |  |  |  |
|  |  | D_60%_ < 34.1% |  |  |  |
|  |  | D_80%_ < 17.2% |  |  |  |
|  | Body | D_max_ < 107.0% |  |  |  |
|  |  |  |  |  |  |
| PM breast | Spinal cord | D_max_ < 12.8 Gy |  | CTV | D_95%_ ≥ 95.0% |
|  | Heart | V_25Gy_ ≤ 10.0% |  | Chest wall | D_95%_ ≥ 95.0% |
|  |  | V_20Gy_ ≤ 5.0% |  |  |  |
|  |  | D_mean_ ≤ 6.0 Gy |  |  |  |
|  | Lungs | V_5Gy_ ≤ 60.0% |  |  |  |
|  |  | V_20Gy_ ≤ 20.0% |  |  |  |
|  | Brachial plexus left | D_max_ ≤ 53.0 Gy |  |  |  |
|  | Humeral head left | D_max_ ≤ 40.0 Gy |  |  |  |
|  | Glnd thyroid | V_30Gy_ ≤ 10.0% |  |  |  |
|  | Trachea | D_max_ ≤ 25.00 Gy |  |  |  |
|  | Esophagus | D_max_ ≤ 20.00 Gy |  |  |  |
|  | Body | D_max_ < 110.0% |  |  |  |
|  |  |  |  |  |  |
| gMALT | Spinal cord | D_max_ < 18.0 Gy |  | PTV | D_2%_ < 105.0% |
|  | Kidney right | D_mean_ < 11.00 Gy |  |  | D_95%_ > 96.0% |
|  |  | V_10Gy_ < 8.0% |  |  | D_98%_ > 92.0% |
|  | Kidney left | D_mean_ < 11.00 Gy |  |  |  |
|  |  | V_10Gy_ < 45.0% |  |  |  |
|  |  | V_20Gy_ < 5.0% |  |  |  |
|  | Heart | V_15Gy_ < 5.0% |  |  |  |
|  | Liver | D_mean_ < 14.0 Gy |  |  |  |
|  |  |  |  |  |  |
| Rectum | Bladder | D_max_ < 110.0% |  | PTV | D_2%_ < 110.0% |
|  |  | V_40Gy_ < 40.0% |  |  | D_95%_ > 95.0% |
|  | Bone Marrow | V_40Gy_ < 30.0% |  |  | D_98%_ > 90.0% |
|  |  | V_10Gy_ < 85.0% |  |  |  |
|  | Femoral head right | V_30Gy_ < 40.0% |  |  |  |
|  | Femoral head left | V_30Gy_ < 40.0% |  |  |  |
|  |  |  |  |  |  |
| Lung SBRT | Lung | V_40_ _Gy_ ≤ 100 cm^3^ |  | PTV | 125% ≤ D_2%_ ≤ 130% |
|  |  | D_mean_ < 18.0 Gy |  |  | D_95%_ = 100% |
|  |  | V_15Gy_ ≤ 25.0% |  |  |  |
|  |  | V_20Gy_ ≤ 20.0% |  |  |  |
|  | Spinal cord | D_max_ < 25 Gy |  |  |  |
|  | Esophagus/pulmonary artery | V_40Gy_ ≤ 1 cm^3^ |  |  |  |
|  | Heart | V_30Gy_≤ 15 cm^3^ |  |  |  |
|  | Stomach/intestine | V_36Gy_≤ 10 cm^3^ |  |  |  |
|  |  | V_30Gy_≤ 100 cm^3^ |  |  |  |
|  | Trachea/main bronchus | V_40Gy_ ≤ 10 cm^3^ |  |  |  |
|  | Brachial plexus | V_25Gy_ ≤ 3 cm^3^ |  |  |  |
|  | Other organs | V_48Gy_ ≤ 1 cm^3^ |  |  |  |
|  |  | V_40Gy_ ≤ 10 cm^3^ |  |  |  |

*Abbreviations:* H&N: Head and neck; PM breast: Postmastectomy breast; gMALT: Gastric mucosa-associated lymphoid tissue lymphoma; SBRT: stereotactic body radiotherapy; Dx: The minimum dose delivered to x% of the structure volume; Vx: the volume receiving no less than x% of the prescription dose; PTV: Planning target volume; CTV: Clinical target volume.
